# Acute pancreatitis associated with diabetic ketoacidosis in a child with COVID-19 infection

**DOI:** 10.1186/s12879-023-08371-0

**Published:** 2023-06-07

**Authors:** Xiaoyan Liu, Qingle Yu, Lanying Li, Songqing Wei, Jin Zeng, Jie Jiang

**Affiliations:** The Affiliated Changsha Central Hospital, Department of Pediatrics, Hengyang Medical School, University of South China, Changsha, China

**Keywords:** Acute pancreatitis, Diabetic ketoacidosis, COVID-19, Hypertriglyceridemia, Child patient

## Abstract

**Background:**

There is a mutual influence between COVID-19, diabetes ketoacidosis, and acute pancreatitis, with clinical manifestations overlapping each other, which can lead to misdiagnosis and delayed treatment that could aggravate the condition and affect the prognosis. COVID-19-induced diabetes ketoacidosis and acute pancreatitis are extremely rare, with only four case reports in adults and no cases yet reported in children.

**Case presentation:**

We reported a case of acute pancreatitis associated with diabetic ketoacidosis in a 12-year-old female child post novel coronavirus infection. The patient presented with vomiting, abdominal pain, shortness of breath, and confusion. Laboratory findings showed elevated levels of inflammatory markers, hypertriglyceridemia, and high blood glucose. The patient was treated with fluid resuscitation, insulin, anti-infection treatments, somatostatin, omeprazole, low-molecular-weight heparin, and nutritional support. Blood purification was administered to remove inflammatory mediators. The patient's symptoms improved, and blood glucose levels stabilized after 20 days of admission.

**Conclusion:**

The case highlights the need for greater awareness and understanding of the interrelated and mutually promoting conditions of COVID-19, diabetes ketoacidosis, and acute pancreatitis among clinicians, to reduce misdiagnosis and missed diagnoses.

## Background

COVID-19, also known as coronavirus disease 2019, can trigger diabetes ketoacidosis (DKA) in patients with diabetes mellitus (DM). DKA often presents with abnormalities in blood lipid metabolism, leading to hypertriglyceridemia (HTG), which in severe cases may trigger acute pancreatitis (AP). COVID-19, DKA, and AP are interrelated and mutually promoting, with overlapping clinical features that may result in misdiagnosis or delayed treatment, exacerbating the condition and leading to poor prognosis. Although COVID-19-induced DKA leading to AP is extremely rare, with only four similar cases reported in adults [1–4], this article reports the known first case in a child, highlighting the need for greater awareness and understanding of the disease among clinicians.

## Case report

A 12-year-old female patient was admitted to the hospital due to vomiting, abdominal pain for 1 day, and shortness of breath with consciousness disorder for half a day. She had been vomiting six to seven times a day for the past day, with stomach contents, accompanied by difficult-to-relieve abdominal pain, poor mental state, which gradually worsened to a state of confusion. Moreover, she exhibited deep and heavy breathing, dizziness and chest tightness. No fever, cough, diarrhea or other discomforts were present. She received treatment at a local hospital where blood glucose and serum electrolyte levels were not measured. After about 4 h of receiving “fluid replacement, anti-infection, and omeprazole gastroprotection” treatment, however her symptoms did not improve. The patient was then transferred to our emergency department and had a rapid blood glucose test, which showed a result of 29.8 mmol/L. She was then admitted to our PICU on December 29, 2022. Since the onset of her symptoms, the patient has had poor mental status and appetite, no bowel movement, increased urine output, and no significant change in weight. The patient has had a history of close contact with a confirmed COVID-19 patient who had fever. The patient has received two doses of the Sinovac COVID-19 vaccine and has no history of prior COVID-19 infection. After admission, further inquiry into the medical history revealed that the patient had experienced symptoms of polydipsia (> 2000 ml/d), polyuria (> 2500 ml/d, with 3–4 nighttime urinations), and weight loss (total decrease of 4 kg) over the past six months, but had not received any medical treatment. The patient has no history of pancreatitis. Her father is in good health, while her mother died in 2021. Both her mother and grandmother had a history of diabetes but denied having a history of hyperlipidemia or coronary heart disease.

Admission Physical Examination: Body temperature was 38℃, heart rate was 155 beats/min, respiratory rate was 38 breaths/min, blood pressure was 145/96 mmHg, body weight was 48 kg, oxygen saturation was 97%, and Glasgow Coma Scale score was 12 with a state of confusion and mental fatigue. There were deep and large respirations, dry skin, bilateral pupils of equal size and round shape at approximately 3.0 mm, with sensitive responses to light. The lips were not cyanotic, and there was congestion in the throat. There was no resistance in the neck, coarse breath sounds were heard in both lungs without rales. Heart rate was 155 beats/min, regular, with strong heart sounds and no murmurs. The abdomen was distended, without evidence of abdominal wall varices, and had tenderness throughout the entire abdomen. The liver and spleen were not palpable under the ribs, and bowel sounds were normal at 5 times/min. Muscle tone in all four limbs was normal, and no pathological signs were observed. Limb extremities were warm with a capillary refill time of less than 2 s.

**Fig. 1:**
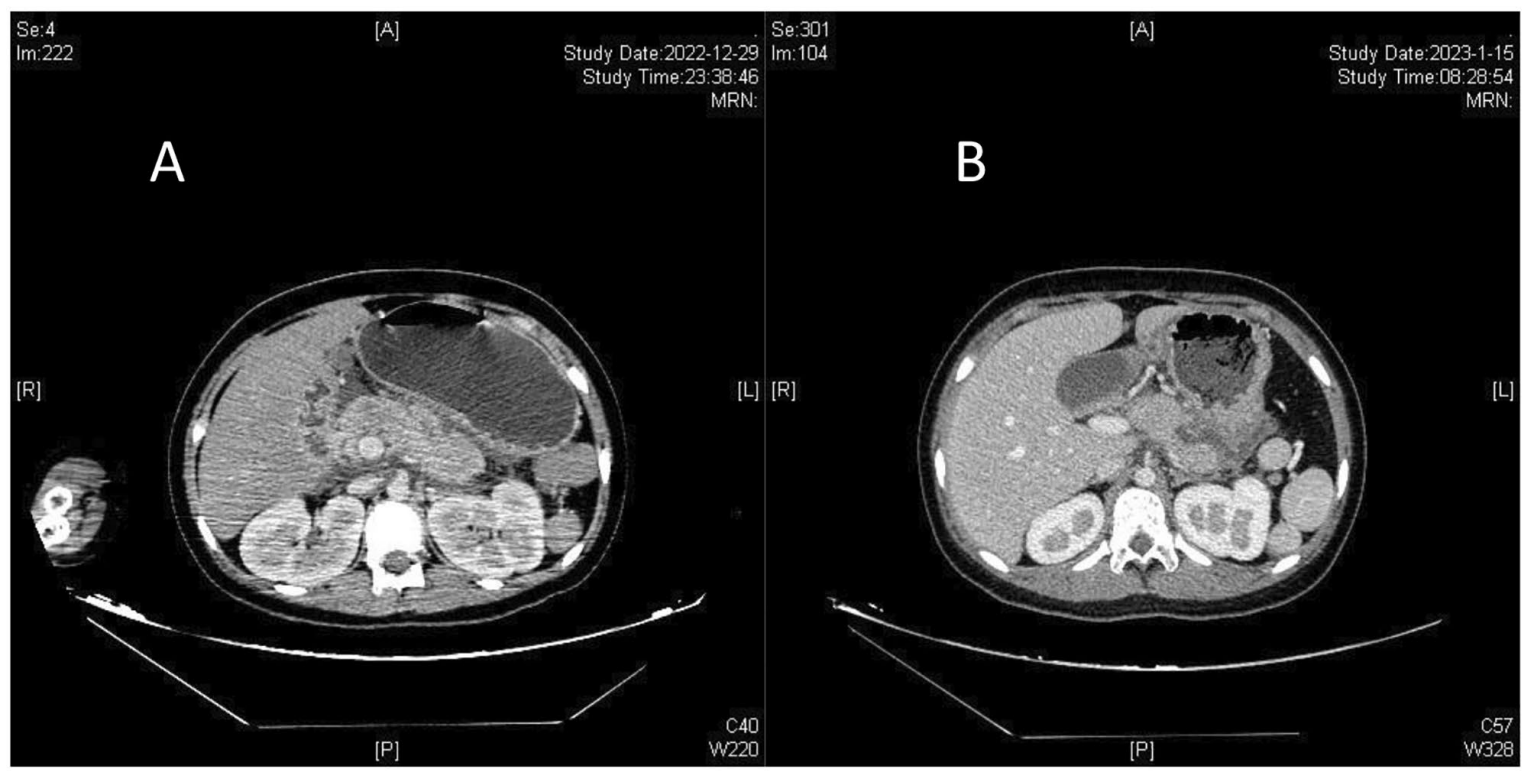
CT of the child’s abdomen. (**A**) Pre-treatment abdominal CT scan with contrast enhancement showed pancreatic swelling with a relatively small pancreatic tail. The pancreatic head partially encircled the descending part of the duodenum, and no abnormal high-density shadows were observed in the substance of the pancreas. The fat gap around the pancreas was murky, and there were scattered exudative shadows. The liver density was decreased, suggesting fatty liver. Multiple nodular shadows with spleen-like density were observed in the splenic area, and they enhanced uniformly after contrast enhancement. (**B**) After 18 days of treatment, the abdominal CT scan with contrast enhancement showed a partial reduction in pancreatic swelling. Irregular strip-like low-density shadows were observed in the pancreatic and gastric interstitial space, and there was no obvious enhancement after contrast. The scattered exudative shadows in the surrounding area were reduced compared to before treatment

Laboratory Findings: The patient’s rapid blood glucose level was measured to be 32.7mmol/L, while the blood gas analysis revealed a pH of 7.01, HCO3 of 4.00mmol/L, BEecf of -27mmol/L, sodium level of 132.0mmol/L, and chloride level of 94.0mmol/L. The urine analysis showed a milky appearance with 3 + levels of ketone bodies and 3 + levels of protein and 4 + levels of glucose. Blood tests showed elevated levels of inflammatory markers such as a white blood cell count of 38.78 × 10^9/L, 80.4% neutrophils, hemoglobin level of 269 g/L, platelets count of 530 × 10^9/L, and a C-reactive protein level of 123.00 mg/L. Additionally, the patient’s calcitonin level was 6.35ng/mL, amylase level was 666U/L in blood, 2123U/L in urine, and lipase level was 1053U/L. The patient had a high total cholesterol level of 9.44mmol/L, triglycerides level of 25.49mmol/L, low-density lipoprotein level of 3.92mmol/L, and a low high-density lipoprotein level of 0.53mmol/L. The glycated hemoglobin level was 16.4%, and the fibrinogen level was 9.4 g/L. The patient’s D-dimer level was 1.8ug/mL, while liver and thyroid function test results were normal. The patient’s uric acid level was 579µmol/L. The patient tested positive for the novel coronavirus nucleic acid (N gene CT value: 25.67; ORF1ab gene CT value: 27.25), but antigen tests for influenza A and B, CMV DNA, EBV DNA, HBV antigen, adenovirus nucleic acid, rotavirus antigen, norovirus nucleic acid, blood culture and stool culture were all negative. The patient tested negative for all five autoantibodies associated with type 1 diabetes and genetic sequencing for diabetes-related genes also returned negative.The C-peptide release test showed a fasting C-peptide level of 0.215nmol/L, 1-hour postprandial C-peptide level of 0.527nmol/L, and 2-hour postprandial C-peptide level of 0.44nmol/L. A chest computed tomography (CT) scan revealed no apparent abnormalities, while the abdominal CT scan showed acute pancreatitis, multidirectional spleen syndrome with partially circular pancreas, and fatty liver (Fig. 1).

Treatment Process and Prognosis: Upon admission, the severity of dehydration in the patient was immediately evaluated and vital signs, blood glucose, consciousness level, pupils, urine output were monitored. The patient was kept on a fasting regimen and received fluid resuscitation. Small doses of insulin were continuously administered through intravenous drip to control blood glucose levels, with an initial dose range of 0.05–0.1 U/(kg·h). Blood glucose levels were monitored hourly, and the rate of blood glucose reduction was controlled at 2–5 mmol/L per hour. The intravenous drip rate was adjusted according to blood glucose and urine output. In response to acute pancreatitis, the patient was given fasting, anti-infection treatments, continuous infusion of somatostatin to suppress pancreatic fluid and enzyme secretion, omeprazole to inhibit acid production, low-molecular-weight heparin to prevent coagulation, nutritional support, and maintenance of organ function with the goal of comprehensive treatment. On the second day after admission, due to the patient’s systemic inflammatory response syndrome, poor correction of acidosis, fever, significantly elevated CRP and procalcitonin, and concurrent novel coronavirus infection, continuous veno-venous hemodiafiltration was administered to remove inflammatory mediators and maintain internal stability. After 12 h of treatment, the patient’s consciousness became clear, and acidosis was corrected. On the fourth day after admission, blood β-hydroxybutyrate levels returned to normal, urine ketones turned negative, and serum lipase, amylase, and triglyceride levels decreased significantly. The patient was given low-fat enteral nutrition powder via gastrostomy tube. On the eighth day of admission, the nucleic acid test for the novel coronavirus has turned negative, the patient’s abdominal pain symptoms improved, and blood lipid, amylase, and lipase levels returned to normal. The patient was switched to a self-administered diet for diabetes and given subcutaneous insulin injections while adjusting the insulin dose based on blood glucose levels. On the twentieth day of admission, the patient’s blood glucose levels stabilized, and re-examination of the abdomen with CT showed significant improvement in pancreatic edema with the presence of a slight encapsulated necrosis in the pancreatic gastric space (Fig. 1). The patient was discharged with a diagnosis of severe diabetic ketoacidosis, type 1 diabetes, moderate to severe acute pancreatitis, hypertriglyceridemia, systemic inflammatory response syndrome, novel coronavirus infection, and multisplenosis. The patient continued subcutaneous insulin therapy at home and was scheduled for regular follow-up visits. Changes in laboratory indicators of the patient upon admission are shown in Table 1.

**Table 1:** Dynamic Monitoring Results of Laboratory Examinations of Affected Children

Laboratory Examination Index	Admission	Time Day 2	Time Day 4	Time Day 8	Time Day 19	Reference Range
White Blood Cell Count (×10^9/L)	38.78	20.99	8.85	7.67	5.27	4.3–11.3
C-reactive Protein (CRP) (mg/L)	123	166.55	93.5	11.3	< 0.5	< 10
Procalcitonin (PCT) (ng/mL)	1.49	6.35	2.01	0.13	< 0.05	< 0.05
Amylase (U/L)	666	384	89	84	—	35–135
Lipase (U/L)	1053	586	71	67	—	1–60
Urine Amylase (U/L)	—	—	2123	326	—	< 450
Triglycerides (mmol/L)	25.49	9.27	3.46	—	2.55	0.55–1.7
Total Cholesterol (mmol/L)	9.44	5.21	4.44	—	5.88	3.4–5.2
High-density Lipoprotein (HDL) (mmol/L)	0.53	0.48	0.39	—	0.8	0.78-2.0
Low-density Lipoprotein (LDL) (mmol/L)	3.92	1.54	2.4	—	4.12	< 2.6
β-Hydroxybutyrate (mmol/L)	6.83	2.78	0.32	—	—	0.03–0.3
Ketone Bodies	3+	3+	Negative	Negative	Negative	Negative
pH	7.01	7.2	7.45	7.47	—	7.35–7.45
HCO3- (mmol/L)	4	8	24	23	—	22–27

## Discussion

The interrelatedness between COVID-19, DKA, and AP remains unclear, and their mechanisms of onset are yet to be determined. Evidence suggests that the pancreas is one of the targeted organs for the SARS-CoV-2 virus, as it can enter cells through ACE2 receptors on the surface of pancreatic islet cells, leading to impaired pancreatic function [5]. In addition to direct damage, SARS-CoV-2 can also induce autoimmune reactions, resulting in β-cell dysfunction and reduced insulin release, and increased insulin resistance in peripheral tissues, leading to the onset or exacerbation of diabetes mellitus (DM) [6]. Therefore, COVID-19 is likely to cause abnormal blood glucose fluctuations in children with DM, leading to the onset of DKA and even life-threatening conditions.There is still controversy about whether new-onset diabetes triggered by COVID-19 infection can gradually recover with the elimination of the infection. Cromer et al. [7] found that about 41% of newly diagnosed DM in the context of COVID-19 infection regressed to normoglycemia within a year of DM diagnosis, and Patients with newly diagnosed DM had, on average, lower glucose levels and HbA1c on admission than those with pre-existing DM. This phenomenon may be related to acute inflammation and insulin resistance,rather than autoimmunity or direct injury to beta cells. However, in many studies, the incidence of DKA was high in patients with both newly diagnosed DM and pre-existing DM in the context of COVID-19 infection, suggesting that the mechanisms of acute insulin deficiency may also be involved [8, 9]. T1DM is an organ-specific autoimmune disease mediated by T lymphocytes that is induced by environmental factors on a genetic basis. Some viruses, such as enteroviruses, CMV, mumps virus, hepatitis virus, rubella virus, etc., play a critical role as triggers of autoimmunity in the occurrence and development of T1DM. Among them, enteroviruses like Coxsackie virus B have been identified as the prime viral candidates for causing T1DM in humans [10]. Various studies found a significant link between COVID-19 disease and the overexpression of Interleukin-6 (IL-6) [11], while the immune response mediated by IL-6 can induce insulin resistance and injury and apoptosis of pancreatic β-cells [12]. Conversely, diabetes is a risk factor that can exacerbate COVID-19. Studies have shown that the expression levels of ACE2 in the lungs, kidneys, liver, heart and pancreas of diabetic patients are significantly higher than those of healthy individuals, leading to these tissues being more vulnerable to attack by SARS-CoV-2. As a result, diabetic patients face a higher risk of contracting SARS-CoV-2 infection, and are more likely to progress to multi-organ damage [5]. Therefore, there is a bidirectional relationship between COVID-19 and diabetes. The child in this case had symptoms of fever and tested positive for SARS-CoV-2 nucleic acid, and combined with their history of close contact with COVID-19, the diagnosis of COVID-19 was confirmed. The patient had a history of polyuria and polydipsia for half a year, and a high HbA1c level of 16.4% which means she was diabetic but not diagnosed. Therefore, we believe COVID-19 infection may have triggered DKA, and the patient was diagnosed with T1DM according to diagnostic criteria [13]. COVID-19 can cause digestive symptoms such as vomiting, abdominal pain, diarrhea, and anorexia, which are also common initial symptoms of DKA. Thus, for children with COVID-19, blood glucose levels should be actively monitored, and DKA should be quickly identified and treated appropriately.

The occurrence of acute pancreatitis (AP) in this patient may be caused by multiple factors. Firstly, diabetic ketoacidosis (DKA) can induce hypertriglyceridemia (HTG), which in turn can lead to AP. DKA is a state of insulin deficiency, often accompanied by abnormalities in lipid metabolism, which can lead to HTG. Nair et al. [14] found that 11% of adults with DKA had concurrent AP, while only 2% of pediatric DKA patients had AP, possibly due to the significantly higher incidence of HTG in adults compared to children. The risk of AP occurrence is closely related to the level of triglycerides (TG), with a significantly increased risk of AP in DKA patients with TG levels above 11.1 mmol/L, while the risk of AP is reduced when TG levels are below 5.65 mmol/L [15]. In this case, the patient had milky blood and urine, and a high blood TG level of 25.49 mmol/L, indicating a high possibility of AP caused by HTG. However, the specific mechanism of AP caused by HTG is not yet fully understood, but may be related to the cellular damage, edema, and ischemia induced by the breakdown of free fatty acids from triglycerides, as well as pancreatic circulation disorders caused by high chylomicronemia [16]. Secondly, COVID-19 can directly lead to the occurrence of AP. SARS-CoV-2 can cause direct damage to both exocrine and endocrine cells, leading to pancreatitis, and can also cause pancreatitis through cytokine storm-mediated pancreatic injury [17]. In addition, severe COVID-19 can cause severe diffuse mucosal subvascular endothelial inflammation leading to diffuse microischemic lesions, pancreatic hypoperfusion, and ischemic injury [18]. The patient in this case had a COVID-19 infection, and other common pathogen tests were negative, indicating that SARS-CoV-2 infection may have contributed to the occurrence of AP. Thirdly, the patient had multiple spleen syndrome with circular pancreas and short pancreatic tail, which may affect pancreatic secretion and increase the risk of AP under other influencing factors.

Patients with the triad of DKA, severe HTG, and AP have a higher incidence of multiple organ dysfunction, parenteral nutrition requirements, and longer hospital stays, with a higher risk of mortality [19]. Therefore, early diagnosis and timely treatment are crucial. However, diagnosing AP caused by HTG induced by DKA may be challenging. Firstly, abdominal pain is a common symptom of DKA, which may mask the coexistence of AP; secondly, about 25% of DKA patients have elevated levels of serum amylase and lipase without clinical or imaging signs of AP, leading to overdiagnosis; In addition, the pancreatic enzyme levels of some patients with DKA combined with AP may remain normal, leading to missed diagnosis.

In conclusion, COVID-19 can induce DKA and AP through direct or indirect effects, and patients with DKA may also have HTG-induced AP, necessitating increased awareness and ability to diagnose and treat this disease, with timely monitoring of blood glucose, lipids, pancreatic enzymes, and abdominal imaging to prevent misdiagnosis and late-stage treatment, leading to improved prognosis.

## Data Availability

The datasets generated and/or analysed during the current study are not publicly available due to concerns regarding patient privacy, but are available from the corresponding author on reasonable request.
